# A Novel and Highly Selective Epidermal Growth Factor Receptor Inhibitor, SMUZ106, for the Treatment of Glioblastoma

**DOI:** 10.3390/pharmaceutics15051501

**Published:** 2023-05-15

**Authors:** Ying Jiang, Chunhui Huang, Yaqi Huang, Lifan Long, Guowu Wu, Fengqiu Guo, Chuan Huang, Siming Liu, Zhengguang Zhu, Shaoyu Wu, Zhonghuang Li, Jiajie Zhang, Shanhe Wan

**Affiliations:** Guangdong Provincial Key Laboratory of New Drug Screening, School of Pharmaceutical Science, Southern Medical University, Guangzhou 510515, China; jy691230@i.smu.edu.cn (Y.J.); h22020266@i.smu.edu.cn (C.H.); yangzichao33@smu.edu.cn (L.L.); guofengqiu@i.smu.edu.cn (F.G.); hc19970707@i.smu.edu.cn (C.H.); liusiming@smu.edu.cn (S.L.); zgg@smu.edu.cn (Z.Z.); wushaoyu@smu.edu.cn (S.W.); lzhuang@fimmu.com (Z.L.)

**Keywords:** SMUZ106, epidermal growth factor receptor (EGFR), glioblastoma (GBM), EGFRvIII, temozolomide-resistance

## Abstract

Targeting the epidermal growth factor receptor (EGFR) is one of the potential ways to treat glioblastoma (GBM). In this study, we investigate the anti-GBM tumor effects of the EGFR inhibitor SMUZ106 in both in vitro and in vivo conditions. The effects of SMUZ106 on the growth and proliferation of GBM cells were explored through MTT and clone formation experiments. Additionally, flow cytometry experiments were conducted to study the effects of SMUZ106 on the cell cycle and apoptosis of GBM cells. The inhibitory activity and selectivity of SMUZ106 to the EGFR protein were proved by Western blotting, molecular docking, and kinase spectrum screening methods. We also conducted a pharmacokinetic analysis of SMUZ106 hydrochloride following *i.v.* or *p.o.* administration to mice and assessed the acute toxicity level of SMUZ106 hydrochloride following *p.o.* administration to mice. Subcutaneous and orthotopic xenograft models of U87MG-EGFRvIII cells were established to assess the antitumor activity of SMUZ106 hydrochloride in vivo. SMUZ106 could inhibit the growth and proliferation of GBM cells, especially for the U87MG-EGFRvIII cells with a mean IC_50_ value of 4.36 μM. Western blotting analyses showed that compound SMUZ106 inhibits the level of EGFR phosphorylation in GBM cells. It was also shown that SMUZ106 targets EGFR and presents high selectivity. In vivo, the absolute bioavailability of SMUZ106 hydrochloride was 51.97%, and its LD_50_ exceeded 5000 mg/kg. SMUZ106 hydrochloride significantly inhibited GBM growth in vivo. Furthermore, SMUZ106 inhibited the activity of U87MG-resistant cells induced by temozolomide (TMZ) (IC_50_: 7.86 μM). These results suggest that SMUZ106 hydrochloride has the potential to be used as a treatment method for GBM as an EGFR inhibitor.

## 1. Introduction

Glioblastoma (GBM) is the most common and aggressive malignant primary brain tumor, accounting for 45.6% of all malignant brain tumors in the world [1,2]. Due to the special location of glioma growth, surgical treatment is challenging, making it difficult for surgeons to completely remove. Thus, radiotherapy and chemotherapy are the standard treatments used for patients with the disease [3]. The chemotherapy drug mainly used in the field is the DNA alkylating agent temozolomide (TMZ), which has improved the median survival rate to 14.6 months [4,5]. However, TMZ is toxic to patients, and reports of patients being clinically resistant to it exist in the literature [6]. Despite the aggressive treatment measures, GBMs invariably continue to grow. Therefore, an extensive search for new and targeted therapies, such as protein kinase inhibitors and immunotherapy, is being conducted [7].

The majority of GBMs have been identified as involving activation of the receptor tyrosine kinase (RTK) signaling pathway. In this context, the epidermal growth factor receptor (EGFR) pathway is one of the most relevant [8]. It has been reported that EGFR amplifications and/or mutations could promote cell proliferation and survival processes in primary GBMs [9]. EGFR mutations mainly occur and cluster in the extracellular (EC) domain and include in-frame deletions (such as the common “variant III”), which have been detected in over 25% of GBM tumors [10]. Therefore, the use of EGFR inhibitor to treat GBM patients have been considered a very attractive approach. First-, second-, and third-generation EGFR inhibitors, such as gefitinib, afatinib, and Osimertinib, have been proven to inhibit GBM cell growth and proliferation [11]. However, gefitinib and afatinib have not shown therapeutic efficacy in clinical trials, due to their poor penetration of the blood–brain barrier [12,13]. Although Osimertinib has a significant brain penetration ability and has been used in phase I/II trials for the treatment of GBMs [14,15,16], the anti-EGFRvIII activity of the drug remains to be improved [2]. In summary, developing novel EGFR inhibitors with a high inhibitory activity against the EGFRvIII mutation in GBMs is a potential direction forresearch.

Fortunately, our research group synthesized 6-(1-(piperidin-4-yl)-1H-pyrazol-4-yl)-N-(quinolin-6-yl)quinazolin-4-amine (compound SMUZ106). SMUZ106 is a novel small-molecule tyrosine kinase inhibitor that inhibits the kinase activity of EGFR kinase (IC_50_ value of 44.1 nM), and it also presents good activity against different tumor cells [17]. Our study shows that SMUZ106 hydrochloride possesses high absolute bioavailability and pharmaceutical properties and low acute toxicity levels. Based on the excellent druggability of SMUZ106, the aim of this study is to demonstrate the targeting and selectivity of SMUZ106 to EGFR and to investigate whether SMUZ106 can be used to overcome TMZ resistance, treating EGFRvIII overexpression in GBMs and effectively inhibiting the proliferation of GBM cells in vivo.

## 2. Materials and Methods

### 2.1. Materials

Compounds SMUZ106 and SMUZ106 hydrochloride (purity > 98%) were combined at the Guangdong Provincial Key Laboratory of New Drug Screening, School of Pharmaceutical Science, Southern Medical University (Guangzhou, China). Gefitinib and temozolomide (TMZ) were obtained from Macklin (Shanghai, China), solubilized in DMSO, and stored at −20 °C. MTT was obtained from Biofroxx (Eisenach, Germany). The following antibodies were procured from Cell Signaling Technology (Danvers, MA, USA): anti-phospho-EGFR (Y1173) (4407s), anti-EGFR (4267s), anti-phospho-ERK (Thr202/Tyr204) (4370s), and anti-phospho-AKT (Ser473) (4060s). Additionally, anti-GAPDH (FD0063) was procured from Ford Biotechnology Co., Ltd. (Hangzhou, China). To perform the Western blotting analyses, the above-mentioned antibodies were used in a dilution of 1:1000. The Cell Cycle and Apoptosis Analysis Kit (Cat. number: C1052) and Annexin V-FITC Apoptosis Detection Kit (Cat. number: C1062M) were purchased from Beyotime (Shanghai, China).

### 2.2. Cell Lines and Culture Conditions

U87MG and U251 cell lines were obtained from Cellcook (Guangzhou, China) and cultured in cell culture flasks containing DMEM medium with 10% fetal bovine serum (FBS) at 37 °C and 5% CO_2_. U87MG-EGFRvIII cells were gifted to us by Prof. Xingmei Zhang and grown in DMEM supplemented with 10% FBS and 200 μg/mL G418 (Sigma-Aldrich, St. Louis, MO, USA) [18]. U87MG-TMZR cells were cultured in DMEM medium with 10% FBS and 400 μM TMZ at 37 °C and 5% CO_2_.

### 2.3. Cell Viability Assay

U87MG, U251, U87MG-EGFRvIII, and U87MG-TMZR cells were cultured in DMEM supplemented with 10% FBS and plated at low density in 96-well plates (2000 cells per well). Then, the cells were treated with different concentrations of gefitinib or compound SMUZ106 for a period of 72 h. MTT (5 mg/mL) was added to each well, and the cells were incubated for another 4 h at 37 °C. The optical density value at 570 nm was measured using a microplate reader (TECAN, Männedorf, Switzerland).

### 2.4. Colony Formation Assay

U87MG, U251, U87MG-EGFRvIII, and U87MG-TMZR cells were trypsinized, and suspended in 6-well plates. After a period of 24 h, the cells were exposed to compound SMUZ106 with different concentrations (1, 3, and 10 μM), and the U87MG-TMZR cells were exposed to compound SMUZ106 with different concentrations (3, 10, and 30 μM) for 72 h. After 10–14 days of cultivation, the cells were stained with 0.4% trypan blue to visualize the formed colony and to count the number of colonies by Enzyme-Linked Immunospot Analyzer (CTL, Shaker Heights, OH, USA).

### 2.5. Cell Cycle and Apoptosis Analysis

U87MG-EGFRvIII cells were seeded into 6-well plates and treated with different concentrations of the compound SMUZ106 (2.5, 5, and 10 μM) for 48 h. Following the treatment, the cells were harvested, washed twice with cold PBS, and fixed overnight at 4 °C in 70% ethanol, then stained with PI solution to analyze the cell cycle distribution via flow cytometry. The results were determined using the ModFit LT 5.0 software. Each assay was performed in triplicates.

To perform the apoptosis analysis, after being washed with PBS, the cells were resuspended in 195 μL Annexin V-FITC binding solution, and then 5 µL of Annexin V-FITC was added, followed by 10 µL PI. Following incubation at room temperature for 20 min in the dark, cell apoptosis rates were detected by flow cytometry. The data were analyzed using FlowJo_V10 software.

### 2.6. Western Blotting

The GBM cells were exposed to compound SMUZ106 at different concentrations (2.5, 5, and 10 μM) for 48 h, and the total proteins were extracted from the cells using RIPA lysis buffer. The concentration of the obtained protein was determined using the BCA Protein Assay Kit (Thermo Fisher, Waltham, MA, USA). Equivalent protein samples (30 μg) were subjected to SDS-PAGE gels and transferred to NC membranes (PALL, Port Washington, NY, USA). Then, the membranes were blocked with 5% milk in TBST for 1 h at RT and probed with primary antibodies diluted overnight at 4 °C, followed by incubation with appropriate secondary antibodies for 1 h at RT. Finally, the protein bands were visualized and scanned using an imaging system (ProteinSimple, Santa Clara, CA, USA).

### 2.7. Molecular Modeling

A docking study was performed on the Glide module of Schrodinger suite (Maestro 11.1). The crystal structures of EGFR^WT^ (PDB code:4WKQ) were input from the protein data bank (http://www.rcsb.org/ (accessed on accessed on 1 August 2019)) into Maestro Protein Preparation Wizard. The structure of compound SMUZ106 was constructed in Maestro and processed and optimized further with the LigPrep panel. The Extra-Precision (XP) docking mode was considered in the docking process, and other parameters were set by default. The MD simulations performed by Amber12 program refer to our previous research [19,20], and the detailed method used is described in the Supplementary Materials.

### 2.8. Kinase Inhibitory Activity

IC_50_ determinations for EGFR were performed with the Z’-LYTE technology platform (Life Technologies), according to the manufacturer’s instructions. The enzyme activity assays of compound SMUZ106 were performed at a single-point concentration (1 μM) against 76 kinases by using the HTRF KinEASE-TK assay according to the manufacturer’s instructions. 

### 2.9. Pharmacokinetics Study

A total of 72 male Kunming mice with an average weight of 20 g were purchased from Southern Medical University (Guangzhou, China) and divided into three groups, with three mice in each group. SMUZ106 was dissolved in 0.5% CMC-Na to prepare a suspension for oral administration (100 mg/kg). SMUZ106 hydrochloride was dissolved in a saline solution and administered to the mice, either intraperitoneally (25 mg/kg) or orally (100 mg/kg). For the intravenous and oral administration, blood samples (0.5 mL) were collected from the mice’s orbital veins and placed into heparinized tubes at 0.25, 0.5, 1, 2, 6, 8, 12, and 24 h following administration. After centrifuging all the samples at 8000 r/min for 5 min, the supernatants were stored at −80 °C until analysis. Subsequently, the plasma samples were transferred to a clean 1.5 mL centrifuge tube and spiked with 100 μL of the internal standard solution (gefitinib, 1.0 μg/mL). Methyl alcohol was then added to the mixture, vortexed for 2 min, and centrifuged at 13,000 r/min for 30 min. The supernatant was collected and evaporated to dryness at RT. The residue was reconstituted in 300 μL of mobile phase (methyl alcohol: ammonium acetate), sonicated for 1 min, vortexed for 2 min, and then centrifuged at 13,000 r/min for 30 min. A 10 µL aliquot of each sample was injected into the LC-MS/MS system for analysis. The pharmacokinetic parameters, such as half-life (t_1/2_), time of peak plasma concentration (T_max_), maximum plasma concentration (C_max_), mean residence time (MRT), area under the concentration–time curve (AUC), and bioavailability (F), were determined using DAS 2.0 software.

### 2.10. Xenograft Model and Intracranial Injection

The nude BALB/c male mice (18–22 g) were obtained from the Laboratory Animal Center of Southern Medical University (Guangzhou, China). The animal protocols were approved by the Institutional Animal Care and Use Committee of Southern Medical University. A total of U87MG-EGFRvIII cells (1 × 10^7^) were subcutaneously injected into the right back flank of the nude mice for 10 days. The animals were divided randomly into five groups and SMUZ106 hydrochloride (75, 100, and 150 mg/kg/d), gefitinib (100 mg/kg/d), or saline solution were orally administered for 3 weeks. Tumor volumes were measured on every other day and the body weights of mice were measured daily.

Through an intraperitoneal injection of sodium pentobarbital, the nude mice were anesthetized and then immobilized. U87MG-EGFRvIII luciferase-transfected cells (5 × 10^5^ in 5 μL of PBS) were injected to a depth of 3 mm using a Hamilton syringe with a 26-gauge needle. After five days, the tumor growth rate was monitored using bioluminescence imaging in an In-Vivo MS FX PRO (Bruker, MA, USA), subsequent to an injection of luciferin (200 μL of 15 mg/mL) into the mice. Based on the tumor volume, the mice were randomized into two groups; the choice of vehicle or SMUZ106 hydrochloride (300 mg/kg/d) was administered by oral gavage. Additionally, the tumor volume was measured and calculated using Bruker MI SE 7.2 software.

### 2.11. Statistical Analysis

The data collected from three independent experiments are presented as mean ± standard deviation (S.D). For all the statistical analyses, GraphPad Prism 7.0 software was used, and *p* < 0.05 was considered statistically significant. Student’s *t*-tests or one-way ANOVAs were used to determine the statistical probabilities. 

## 3. Results

### 3.1. Effect of SMUZ106 on the Growth and Proliferation of GBM

**Figure 1:**
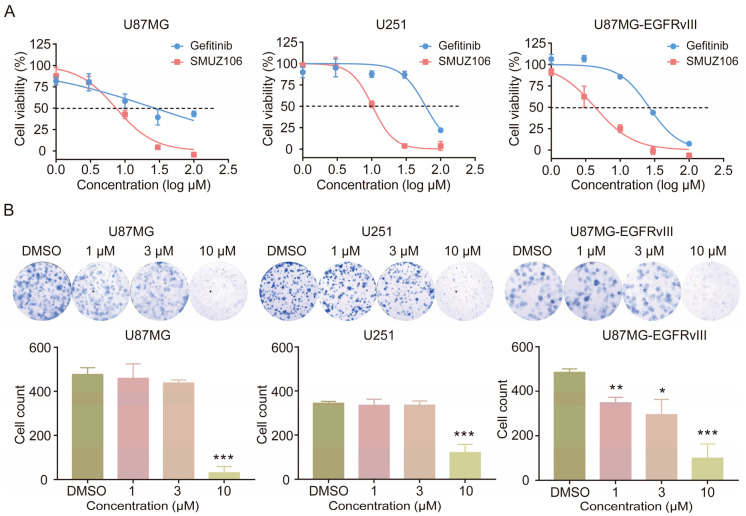
(**A**) The survival curves of GBM cells with the treatment of compound SMUZ106 for 72 h. (**B**) The colony formation of GBM cells and quantitatively analyzed colony number with the treatment of compound SMUZ106. Statistical values represent the mean ± S.D. of three independent experiments, (*) *p* < 0.05, (**) *p* < 0.01, (***) *p* < 0.001 compared to the control group (DMSO).

We measured the proliferation rates of U87MG, U251, and U87MG-EGFRvIII cells treated with gefitinib or compound SMUZ106 using MTT assay. A dose-dependent decrease in the cell viability of GBM cells was observed following gefitinib or compound SMUZ106 treatments. Among them, compound SMUZ106 showed a significantly improved antitumor activity compared with gefitinib. The mean inhibitory concentration values of the 50% (IC_50_) value for compound SMUZ106 were 7.43, 10.29, and 4.36 μM for U87MG, U251, and U87MG-EGFRvIII cells, respectively (Figure 1A). 

Following the treatment with three concentrations (1, 3, and 10 μM) of compound SMUZ106, the number of colonies formed by GBM cells decreased significantly, compared to that in the vehicle-treated control (Figure 1B). The colony formation assay revealed that compound SMUZ106 significantly decreased the clonogenicity of U87MG, U251, and U87MG-EGFRvIII cells. Overall, compound SMUZ106 effectively inhibited the growth of GBM cells. 

### 3.2. Effect of SMUZ106 on Cell Cycle Progression in GBM Cell Lines

**Figure 2:**
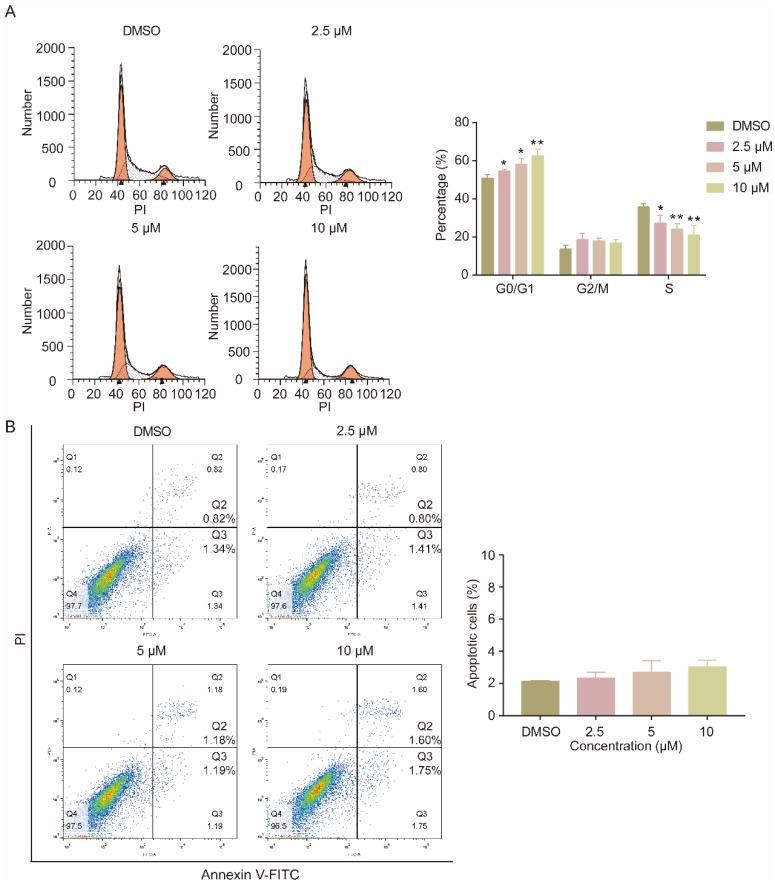
(**A**) Flow cytometry analysis of the cell cycle distribution and the percentage of U87MG-EGFRvIII cells in different phases of the cell cycle with the treatment of compound SMUZ106 for 48 h. (**B**) Annexin V-FITC and PI staining showing apoptotic cells with the treatment of compound SMUZ106 for 48 h. Statistical values represent the mean ± S.D. of three independent experiments, (*) *p* < 0.05, (**) *p* < 0.01, compared to the control group (DMSO).

Furthermore, the effect of compound SMUZ106 on the cell cycle and apoptosis in U87MG-EGFRvIII cells was examined by flow cytometry. The cell cycle analysis demonstrated that compound SMUZ106 significantly increased the percentage of U87MG-EGFRvIII cells in the G0/G1 phase and decreased their quantity cells in the S phase (Figure 2A). Following the treatment with three concentrations of compound SMUZ106 for 48 h, the G0/G1 phase of U87MG-EGFRvIII cells increased from 50.82% to 54.44%, 58.17%, and 62.50%, respectively. The S phase of U87MG-EGFRvIII cells decreased from 35.68% to 27.08%, 24.01%, and 20.78%, respectively. Additionally, we explored the apoptosis effect of compound SMUZ106, and the apoptosis rate of cells was detected by flow cytometry. The results showed that the apoptosis of U87MG-EGFRvIII cells was not obvious at the three concentrations of compound SMUZ106 for 48 h, and the percentages of apoptotic cells were 2.34%, 2.70%, and 3.02%, respectively (Figure 2B). Our study showed that compound SMUZ106 affected the proliferation of U87MG-EGFRvIII cells, mainly by blocking the cell cycle in the G0/G1 phase, rather than by inducing apoptosis.

### 3.3. Effect on the Intracellular Signaling Pathway by the Treatment of SMUZ106 in GBM Cell Lines 

**Figure 3:**
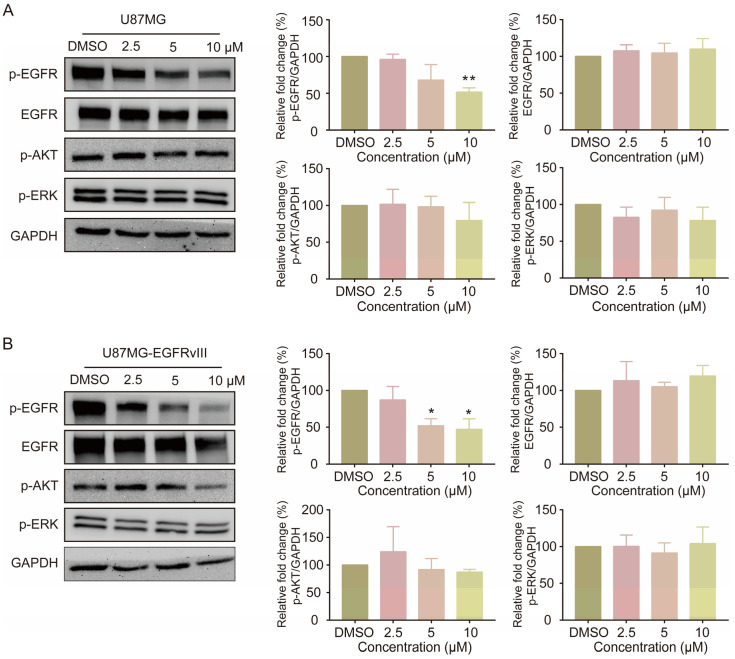
(**A**) U87MG cells and (**B**) U87MG-EGFRvIII cells were treated with the three concentrations of SMUZ106 or DMSO for 48 h. Statistical values represent the mean ± S.D. of three independent experiments, (*) *p* < 0.05, (**) *p* < 0.01, compared to the control group (DMSO).

We treated GBM cells with various concentrations of compound SMUZ106 and analyzed the effects on phospho-EGFR (Tyr1068) and downstream signaling by Western blotting. The protein level of phospho-EGFR (Y1086) decreased dose dependently, while the protein expression level of total EGFR remained unchanged in both U87MG and U87MG-EGFRvIII cells (Figure 3). SMUZ106 treatment did not significantly downregulate the phosphorylation levels of AKT and Erk1/2, which suggests that SMUZ106 may affect other pathways regulated by EGFR. 

### 3.4. Interaction Details of Compound SMUZ106 Binding to EGFR and Kinase Inhibitory Profile

**Figure 4:**
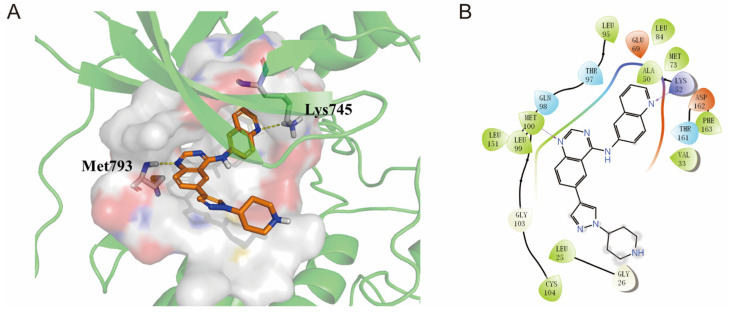
(**A**) Conformation overlay of the binding of compound SMUZ106 to EGFR. (**B**) Conformational 2D image of EGFR interacting with compound SMUZ106.

Molecular dynamics simulations showed that SMUZ106 stably bound to the ATP pocket of EGFR (Figure 4). The quinazoline ring of SMUZ106 formed a hydrogen bond with the backbone of the hinge area Met793 of EGFR, which is the main characteristic of most kinase inhibitors targeting the ATP site. The quinolone ring of SMUZ106 occupied the hydrophobic pocket and interacted with Lys745 via the hydrogen bond, while the piperidine part extended to the solvent area. The binding free energy of SMUZ106 in EGFR was estimated by MMGBSA methods to be −18.85 kcal/mol (Table S1). Both the binding conformation and binding free energy suggested that compound SMUZ106 presented appropriate affinity binding to EGFR, which is consistent with the kinase inhibitory data (compound 12d, IC_50_: 44.1 nM) [17].

**Figure 5:**
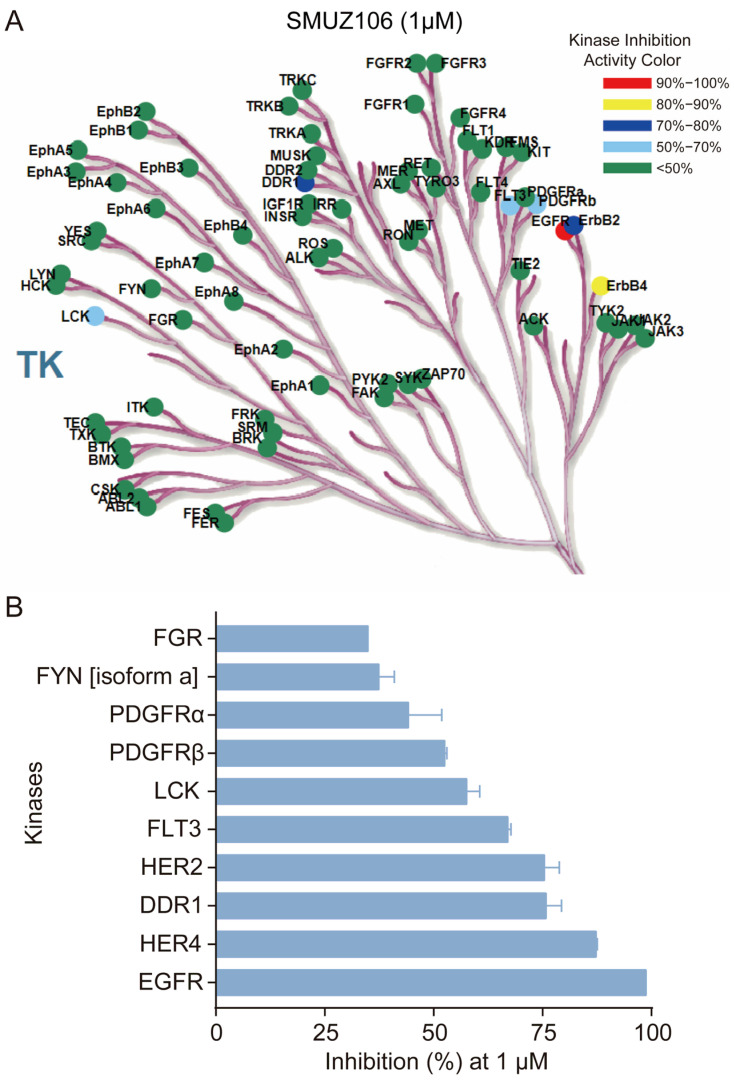
(**A**) Kinase dendrogram of compound SMUZ106 at 1 μM against 76 wild-type kinases by HTRF kinase assay. (**B**) Inhibition rate of compound SMUZ106 against some representative kinases. The experiment was performed by ICE bioscience (Beijing, China).

To explore the broader kinase selectivity, we tested the selectivity of compound SMUZ106 by screening it in a panel of 76 tyrosine kinases using the HTRF KinEASE-TK assay. Consistent with our expectations, compound SMUZ106 exhibited excellent selectivity to EGFR at 1 μM concentration (Figure 5A). Additionally, compound SMUZ106 showed poor inhibitory activity against HER4 and HER2 with percentages of 87.32% and 75.39%, respectively, compared to EGFR (98.73%) (Figure 5B). The data obtained from previous articles by our research group demonstrated that the IC_50_ values of the compound for HER2 and HER4 kinases were 874 and 930 nM, respectively (Figure S1) [17]. Therefore, the results show that compound SMUZ106 is a novel and highly selective EGFR inhibitor.

### 3.5. Pharmacokinetics Study

**Figure 6:**
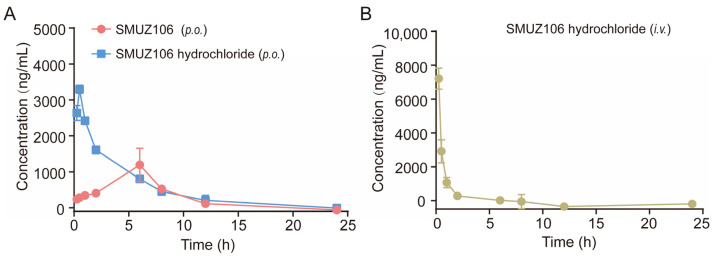
(**A**) The compounds SMUZ106 and SMUZ106 hydrochloride after *p.o.* administration at 100 mg/kg (mean ± SD, *n* = 3). (**B**) SMUZ106 hydrochloride after *i.v.* administration at 25 mg/kg (mean ± SD, *n* = 3).

The solubility of the compound SMUZ106 and its related salts in artificial gastric juice, artificial intestinal fluid, and physiological saline were determined by HPLC. The results showed that the solubility of citrate of the compound SMUZ106 was increased significantly. Additionally, the solubility of SMUZ106 hydrochloride was 195.196 mg/mL, and its solubility increased by 12-fold, compared to the compound SMUZ106 (16.774 mg/mL) (Table S2). Therefore, SMUZ106 hydrochloride was selected for the pharmacokinetic analysis. We measured the plasma concentration of SMUZ106 hydrochloride after *i.v.* or *p.o.* administrations to mice. The pharmacokinetic parameters were shown in Figure 6 and Table 1. The results of the pharmacokinetic parameters of SMUZ106 hydrochloride after *i.v.* administration were as follows: t_max_ = 0.25 h; t_1/2_ = 0.68 ± 0.46 h; C_max_ = 7217.33 ± 624.60 μg/L. After oral administration, the t_max_ of SMUZ106 hydrochloride (0.50 h) was shorter than that of the compound SMUZ106 (6.00 h). The absolute bioavailability values of the compound SMUZ106 and SMUZ106 hydrochloride were 28.96% and 51.97%, respectively. The absolute bioavailability of SMUZ106 hydrochloride was improved by nearly double the amount compared to the compound SMUZ106. In summary, the results show that SMUZ106 hydrochloride presents high absolute bioavailability and certain pharmaceutical properties.

### 3.6. Treatment with SMUZ106 Hydrochloride Suppresses the Growth of U87MG-EGFRvIII In Vivo

**Figure 7:**
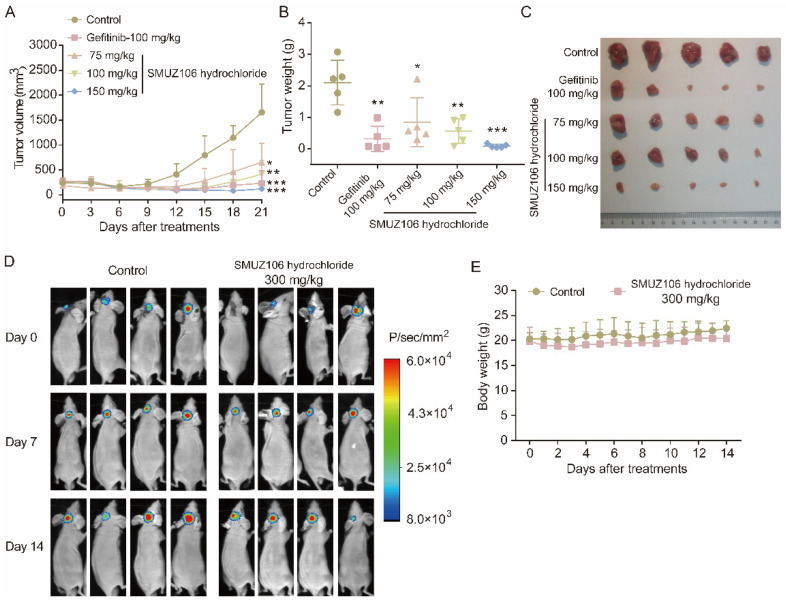
(**A**) The growth curves of tumors on 21 days. (**B**) The tumor sizes after SMUZ106 hydrochloride oral administration for 21 days in a nude mouse subcutaneous tumor model inoculated with U87MG-EGFRvIII cells (*n =* 5). (**C**) The tumor weight changes of nude mice on 20 days. (**D**) U87MG-EGFRvIII luciferase-transfected cells were intracranially injected into mice using a stereotactic frame. The tumor volume was measured using IVIS imaging (*n =* 4). (**E**) The body weight of the mice was measured daily for 14 days. (*) *p* < 0.05, (**) *p* < 0.01, (***) *p* < 0.001 compared to the control group (Solvent group).

Our previous studies have shown that compound SMUZ106 can suppress the proliferation and cell cycle of GBM in vitro. The pharmacokinetic characteristics of SMUZ106 hydrochloride were also evaluated. We also assessed the acute toxicity value of SMUZ106 hydrochloride. The results of the acute toxicity test showed that the LD_50_ value of SMUZ106 hydrochloride exceeded 5000 mg/kg (Table S3), and there was no obvious damage to the main organs of mice following SMUZ106 treatment by H&E staining (Figure S2). To further determine whether compound SMUZ106 can inhibit GBM growth in vivo, we established a subcutaneous and orthotopic xenograft model of U87MG-EGFRvIII cells. In the subcutaneous xenograft experiment, gefitinib was selected as the positive control. The results showed that the compound SMUZ106 and gefitinib were equally effective in inhibiting tumor growth (Figure 7A). The oral administration of SMUZ106 hydrochloride once daily for 21 consecutive days caused a significant tumor growth inhibition effect (Figure 7A), yielding inhibitory rates of 67%, 89%, and 111% in terms of the relative tumor volume at dosages of 75, 100, and 150 mg/kg, respectively. Similar results were observed when measuring the tumor weight on day 21 (Figure 7B,C). In the orthotopic xenograft model, gefitinib was not chosen as the positive control due to poorly penetrated the blood–brain barrier [21]. The results showed a 33% decrease in relative tumor volume was observed at 300 mg/kg/day following 14 days of the oral administration of SMUZ106 hydrochloride (Figure 7D). During the 14 days of treatment, there were no significant differences in the body weight values among the groups of mice (Figure 7E). Collectively, our results suggest that compound SMUZ106 can inhibit the growth of GBM in vivo.

### 3.7. Effect of SMUZ106 on the Growth and Proliferation of U87MG-Resistant Cells Induced by TMZ

**Figure 8:**
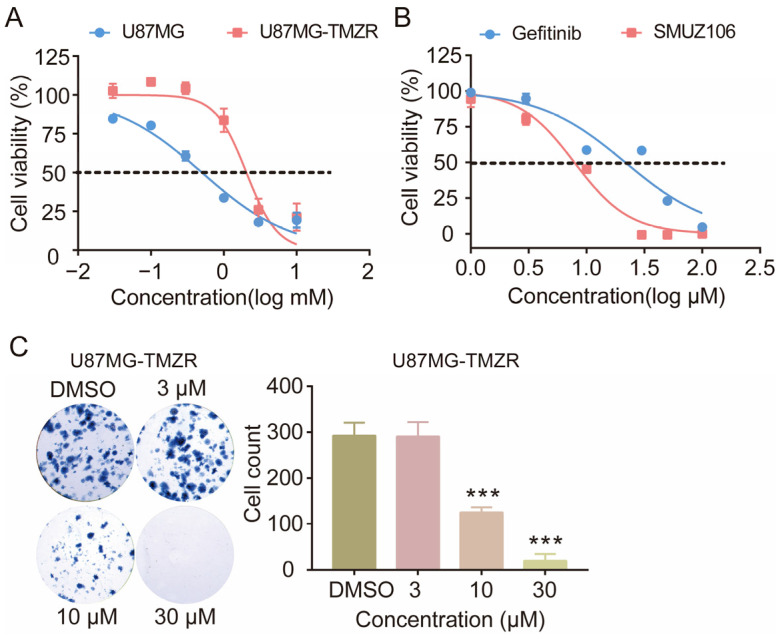
(**A**) The effect of TMZ on the proliferation of U87MG cells and U87MG- TMZR cells. (**B**) The survival curves of U87MG- TMZR cells with the treatment of compound SMUZ106 for 72 h; (**C**) The colony formation of U87MG- TMZR cells and quantitatively analyzed colony number with the treatment of compound SMUZ106. Statistical values represent the mean ± S.D. of three independent experiments, (***) *p* < 0.001 compared to the control group (DMSO).

The emergence of TMZ-resistance behavior limits its anti-GBM activity. The activation of DNA repair pathways is the principal mechanism leading to TMZ resistance; therefore, it was determined that drugs targeting key pathways through different mechanisms can treat TMZ-induced drug-resistant cancer cells [22]. In this study, SMUZ106 was an EGFR inhibitor, which is distinct from the TMZ-resistance pathway. Therefore, we selected U87MG-resistant cells induced by TMZ to evaluate the effect of SMUZ106. The results showed that the inhibitory activity of TMZ on TMZ-resistant U87MG cells (U87MG-TMZR) (IC_50_: 2045 μM) was much greater than that of U87MG cells (IC_50_: 501.4 μM) (Figure 8A). Further experimental results showed that SMUZ106 could inhibit the growth of TMZ-resistant U87MG cells (IC_50_ value of 7.86 μM) (Figure 8B). Similarly, the results obtained for clone formation also showed that SMUZ106 could inhibit the proliferation of TMZ-resistant U87MG cells (Figure 8C). Thus, the EGFR inhibitor SMUZ106 is a potential compound used to overcome TMZ resistance.

## 4. Discussion

The EGFR protein plays an important role in epithelial tissue maintenance. However, the aberrant activation of EGFR signaling can lead to tumorigenesis; therefore, it is one of the most studied tyrosine kinase receptors in the world [23]. EGFR overexpression has been detected in a variety of human tumors, and the amplification of the EGFR gene occurs in approximately 50% of GBM patients [24,25]. Accordingly, EGFR inhibitors are recommended in the research as the first-line treatment for patients with advanced EGFR mutations [26], and the use of these inhibitors for GBM treatment is currently being tested in clinical trials. The first- generation EGFR inhibitor, gefitinib, has shown limited effectiveness due to its poor penetration of the blood–brain barrier (BBB) [21]. Osimertinib, as a third-generation EGFR-TKI, has improved the poor BBB permeability and demonstrated efficacy in the CNS [27]. However, our previous study determined that the inhibitory activity of Osimertinib against U87MG-EGFRvIII cells remains to be improved [19]. It is noteworthy that our experiments showed that compound SMUZ106 can effectively inhibit the proliferation of U87MG-EGFRvIII cells, which is better than gefitinib and Osimertinib. In subcutaneous xenograft experiments, SMUZ106 hydrochloride inhibited tumor growth most significantly at a dose of 150 mg/kg. Taken together, compound SMUZ106 showed outstanding anti-GBM proliferation activity in both in vitro and in vivo experiments.

As an EGFR inhibitor, the MD experiments showed that the binding of compound SMUZ106 to EGFR was stable and specific, and we investigated the ability of SMUZ106 to inhibit EGFR auto-phosphorylation activity in the GBM cells. The results of the kinase spectrum showed that compound SMUZ106 has high selectivity, which is significantly better than afatinib (BIBW2992), lapatinib (GW-572016), and dacomitinib (PF-00299804) [28,29,30]. Studies have also shown that improving the selectivity of drugs can reduce the occurrence of adverse reactions, toxicity, and side effects [31,32]. Moreover, dacomitinib and afatinib were also consistent in causing the most toxicity among EGFR-TKIs, and gefitinib also appeared to be the safest treatment with significant differences when compared to dacomitinib and afatinib [33]. We also evaluated the acute toxicity level of SMUZ106 hydrochloride. The results of acute toxicity test showed that the LD_50_ of SMUZ106 hydrochloride was greater than 5000 mg/kg. According to the five classification criteria of the World Health Organization, SMUZ106 hydrochloride produces no obvious acute toxicity in mice by oral administration.

In addition, TMZ is the most widely used chemotherapeutic drug for the clinical treatment of GBM [34]. However, TMZ resistance is a major problem faced by medics when treating malignant brain tumors [35]. The major resistance mechanisms prevalent are associated with tumor heterogeneity and evolution processes, the abnormal functionality of DNA damage response and DNA repair mechanisms, etc. [36]. The connection evident between the aberrant activation of RTK signaling behavior and the aforementioned resistance mechanisms partially reveals the contribution of TKIs to the treatment of GBMs. Genome-wide studies have identified the aberrant functionality of RTKs as a main feature of GBMs [37] and, among them, EGFR is constitutively activated in approximately 57% of GBMs. Therefore, overcoming the proliferation of drug-resistant cells caused by TMZ can be achieved by inhibiting the activity of EGFR. Combined with the good activity of the EGFR inhibitor SMUZ106 against TMZ-resistant cells presented in this study, it can be suggested that EGFR inhibitors have promising potential for the treatment of GBM resistance in the future.

## 5. Conclusions

In summary, this study reported that the high targeting and selectivity of the EGFR inhibitor SMUZ106 can effectively suppress the growth of U87MG-EGFRvIII cells in both in vitro and in vivo conditions. Additionally, SMUZ106 hydrochloride presents high absolute bioavailability and low toxicity in vivo. The results also show that SMUZ106 can overcome TMZ resistance in vitro. Thus, SMUZ106, as a promising EGFR inhibitor, is expected to be studied further in the treatment of GBM.

## Figures and Tables

**Table 1:** The main pharmacokinetic parameter of compound SMUZ106 and SMUZ106 hydrochloride after administration (mean ± SD, *n =* 3).

Parameters	Unit	SMUZ106 (*p.o.*)	SMUZ106- Hydrochloride (*p.o.*)	SMUZ106- Hydrochloride (*i.v.*)
t_1/2_	h	0.87 ± 0.01	2.66 ± 1.90	0.68 ± 0.46
t_max_	h	6.00	0.50	0.25
C_max_	μg·L^−1^	1193.00 ± 464.06	3297.00 ± 112.58	7217.33 ± 624.60
MRT_0→t_	h	6.32 ± 0.22	4.63 ± 1.23	1.18 ± 1.03
AUC_0→∞_	μg·L^−1^·h	7509.51 ± 1058.57	13,476.20 ± 1782.47	6482.67 ± 952.89
F		28.96%	51.97%	

## Data Availability

The data presented in this study are available on request from the corresponding author.

## Supplementary Materials

The following supporting information can be downloaded at: https://www.mdpi.com/article/10.3390/pharmaceutics15051501/s1. Table S1: Binding free energies of EGFR in complex with the compound SMUZ106 obtained by MM-PBSA and MM-GBSA (kcal/mol); Table S2: The solubility of the compound SMUZ106 and three types of salt at different media; Table S3: The acute toxicity of SMUZ106 hydrochloride after oral administration for 14 days; Figure S1: Biochemical activities (IC50, Mean ± SD, nM) of the compound SMUZ106 on HER2 and HER4; Figure S2: H&E staining of the main organs of mice in the acute toxicity test of SMUZ106 hydrochloride.
