# Supplementary material for: A Novel and Highly Selective Epidermal Growth Factor Receptor Inhibitor, SMUZ106, for the Treatment of Glioblastoma
Source: Pharmaceutics. 2023 May 15;15(5):1501. doi: 10.3390/pharmaceutics15051501 (PMC10220892; doi:10.3390/pharmaceutics15051501)
Supplement: Supplementary file 1 [file pharmaceutics-15-01501-s001.zip › pharmaceutics-2339863-supplementary.pdf]

S1. Supplementary Materials

S1.1 Molecular modeling

Compound SMUZ106 was optimized using Hartree–Fock method 6-31G* basis set in Gaussian 16 1, followed by the RESP method calculating the atomic partial charges 2. AMBER 99SB force fields 3 and General AMBER force field (GAFF) 4 were applied for protein and compounds, respectively. Each complex was placed in the center of a periodic box with a 10 Å buffer size filled with TIP3P water 5 and was neutralized by adding irons. Then all systems were minimized by three steps as follows: (i) restrict the protein-compound complex and minimize the ions and TIP3P water by 4000 steps of steepest descent (SD) minimization and conjugate gradient (CG) minimization; (ii) constraint the backbone atoms of protein and the heavy atoms of compound, minimize others by 4000 steps of SD minimization and 4000 steps of CG minimization; (iii) minimize all substances by 4000 steps of SD minimization and CG minimization without constraint. All systems were heated from 0 to 310 K and went through 500 ps density equilibration, followed by 20 ns MD simulations. The Particle mesh Ewald (PME) method was utilized to calculate electrostatic interactions 6. Hydrogen atoms were restricted by the SHAKE algorithm 7. MMGBSA module in AMBER12 was employed to calculate the binding free energy (ΔGbind) from the last 10 ns MD trajectory with MM-GBSA and MM-PBSA method 8. Among that, 10 structures systematically extracted from the last 5 ns MD trajectory were used to calculate the entropic contribution. PyMOL was used to visualize and analyze the compound-protein complex structures 9.

S2. Supplementary results

Table S1. Binding free energies of EGFR in complex with the compound SMUZ106 obtained by MM-PBSA and MM-GBSA (kcal/mol).

**298K**	**SMUZ106**	**SD**
ΔE_{vdw}	-51.9663	3.1024
ΔE_{ele}	-10.9576	9.9288
ΔE_{MM}	-62.9239	10.4704
ΔE_{PB}	30.4925	9.5359
ΔE_{SA}	-6.0506	0.2785
ΔG_{sol}(GB)	24.4419	9.4642
ΔH_{prep}(GB)	-38.482	2.9759
TΔS	-19.6275	3.4177
ΔG_{prep}(GB)	-18.8545	

Table S2. The solubility of the compound SMUZ106 and three types of salt at different media.

	SMUZ106 (mg/ml)	SMUZ106-hydrochloride (mg/ml)	SMUZ106-hydrobromide (mg/ml)	SMUZ106-Maleate (mg/ml)
normal saline	3.69	194.83	163.96	4.68
artificial gastric juice	16.78	195.20	134.95	20.99
artificial intestinal juice	0.22	135.05	129.79	13.96

Table S3. The acute toxicity of SMUZ106 hydrochloride after oral administration for 14 days (*n = 10*).

Dose (mg/kg)	D/T		Mortality (%)
	female	male	
5000	5/2	5/1	30

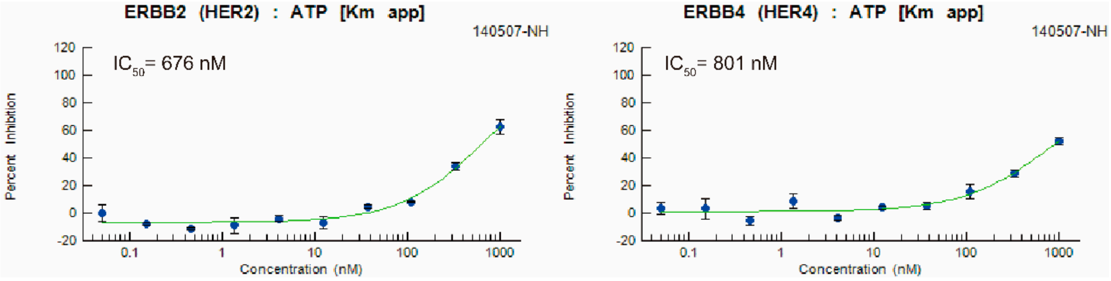

Figure S1. Biochemical activities (IC_{50}, Mean ± SD, nM) of the compound SMUZ106 on HER2 and HER4.

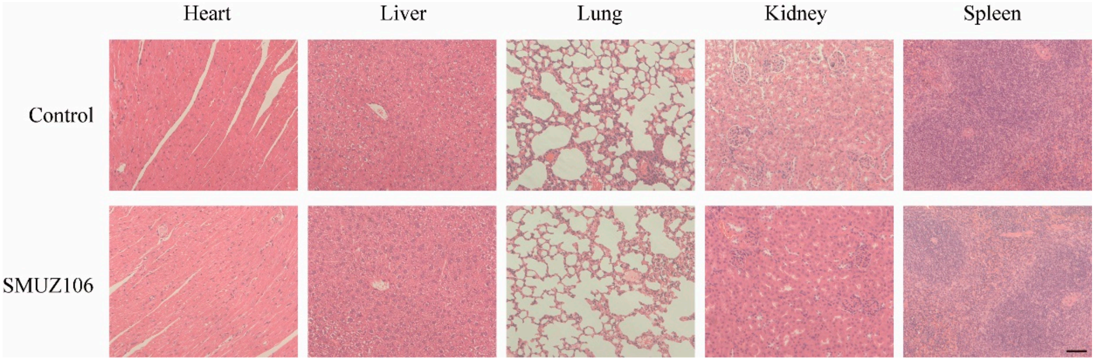

Figure S2. H&E staining of the main organs of mice in the acute toxicity test of SMUZ106 hydrochloride. (Magnification 200×, Scale bar: 50 μm).
